# Analysis of Antimicrobial Residues and Resistance Profiles of *Escherichia coli* and *Enterococcus* spp. in Lagoon Water from California Dairies

**DOI:** 10.3390/vetsci12100960

**Published:** 2025-10-08

**Authors:** Siqi Wang, Sharif S. Aly, Essam Abdelfattah, Pius Ekong, David B. Sheedy, Wagdy ElAshmawy, Betsy M. Karle, Randi Black, Deniece R. Williams, Pramod Pandey, Emmanuel Okello

**Affiliations:** 1 Department of Population Health and Reproduction, School of Veterinary Medicine, University of California, Davis, Davis, CA 95616, USA; 2 Veterinary Medicine Teaching and Research Center, School of Veterinary Medicine, University of California, Davis, Tulare, CA 93274, USA; 3 Department of Animal Hygiene and Veterinary Management, Faculty of Veterinary Medicine, Benha University, Moshtohor 13736, Al Qalyubiyah, Egypt; 4 Cooperative Extension, Division of Agriculture and Natural Resources, University of California, Orland, CA 95963, USA; 5 Cooperative Extension, Division of Agriculture and Natural Resources, University of California, Santa Rosa, CA 95403, USA

**Keywords:** antimicrobial resistance, antimicrobial drug, lagoon water, drug residues, phenotype, *E. coli*, *Enterococcus* spp.

## Abstract

**Simple Summary:**

Antibiotics play a vital role in treating sick animals on dairy farms, but they also contribute to antibiotic resistance, which impacts human, animal, and environmental health. In our study of lagoon water samples from nine California dairy farms, we frequently found residues of antibiotics, including florfenicol, tilmicosin, and tetracycline, while penicillin was much less common. The high florfenicol levels may be attributed to its use in young animals, as it is not approved for adults. We also tested *Escherichia coli* and *Enterococcus* spp./*Streptococcus* spp. (ES) bacteria for antibiotic resistance and observed seasonal, regional, and sampling variability in levels of resistance, though results were not statistically significant. Notably, specific antibiotics showed some associations with bacterial resistance; for instance, tilmicosin residues corresponded with lower *E. coli* resistance to certain antibiotics such as danofloxacin and enrofloxacin, while sulfamethoxazole was linked to increased tetracycline resistance in ES. Overall, our findings highlight the complexity of antibiotic resistance on dairy farms and underscore the need for improved monitoring and prudent antibiotic use to mitigate these risks.

**Abstract:**

The widespread use of antimicrobial drugs (AMDs) in livestock production contributes to antimicrobial resistance (AMR), a global One Health concern affecting humans, animals, and the environment. This study analyzed AMD residues and the AMR profiles in *Escherichia coli* and *Enterococcus* spp./*Streptococcus* spp. (ES) isolated from lagoon water samples collected from nine California dairies. Antimicrobial susceptibility testing was performed using the microbroth dilution method, and enzyme-linked immunosorbent assay (ELISA) kits were used to detect AMD residues in lagoon water. Overall, residues of florfenicol and tilmicosin were detected in more than 90% of the samples, while tetracycline was detected in 74.2 ± 4.6% of the samples. In contrast, penicillin and sulfamethazone residues were low, observed in only 3.4 ± 1.9% and 32.3 ± 5.0% of samples, respectively. The very low prevalence of penicillin was likely due to limited use in dairy cattle, given its prolonged withdrawal period. Prevalence estimates for AMR in the lagoon samples showed 100% resistance of *E. coli* to tiamulin, tilmicosin or tylosin and high prevalence against florfenicol (96.0% ± 2.0) or gamithromycin (92.0% ± 1.9). However, low AMR estimates (less than 10%) were observed against other AMDs tested. Similarly, the prevalence estimates for AMR of ES isolates in the studied lagoon were high against florfenicol (95.1% ± 2.0), tildipirosin (97.6% ± 1.7), or tilmicosin (98.8% ± 1.2), but low against ampicillin (4.9% ± 1.9) and penicillin (8.5% ± 2.4). Despite numerical differences in AMR prevalence by season, region, and sampling point, these variations were not statistically significant. Logistic regression models were applied to explore associations between AMD residues and AMR phenotypes where appropriate. Tilmicosin residues were significantly associated with reduced resistance to danofloxacin, enrofloxacin, and tildipirosin in *E. coli* isolates, while sulfamethoxazole residues were linked to increased tetracycline resistance in *Enterococcus* spp. The presence of florfenicol residues, potentially originating from treated calves and heifers, helps explain the high prevalence of resistance to this drug in both bacterial species. However, not all AMD residues were associated with AMR, underscoring the complex ecological and genetic factors involved in the development and maintenance of resistance in dairy environments. These findings underscore the importance of integrating AMR surveillance and prudent AMD use practices across all segments of dairy production systems.

## 1. Introduction

Antimicrobial drugs (AMD) are crucial for the treatment and control of bacterial infections [1]. However, the widespread use of AMD is associated with an increased risk of antimicrobial resistance (AMR), a global challenge affecting humans, animals, and the environment [2,3]. It is well recognized that the use of antibiotics in humans and animals contributes to the development and spread of antibiotic-resistant bacteria, and the overuse of antibiotics exacerbates this situation [4]. Expression of AMR occurs at two levels: cellular level resistance and community level, such as biofilms [5]. At the cellular level, AMR results from gene mutations or the acquisition of new genetic determinants for resistance from other organisms through horizontal gene transfer. At the community level, AMR occurs because multiple bacteria can endure environmental stress that individual cells cannot, as exemplified by biofilm formation, a structural barrier that protects bacteria from antimicrobial agents. The synergy between cellular and community resistance can significantly enhance the microbial community’s AMR [5].

The use of AMD in livestock for disease treatment or prevention can expose the environment to AMD residues or partially metabolized by-products excreted into water or soil [6,7]. Several studies have reported on AMR in US dairies [8,9,10,11,12,13,14]. However, there are relatively few studies describing the fate of AMD found in stored manure or animal excrement [15]. Moreover, few studies have explored the association between dairy cattle management and their environment on the development of resistance [16,17]. Environmental surveillance for AMR can supplement surveillance for AMR in humans and animals to better understand the risk of its transmission among them [18].

On many free-stall and open-lot dairy farms in California, it is a standard procedure to flush concrete lanes with recycled water [19]. This process helps clean concrete surfaces efficiently and remove any debris. The water used for flushing is then collected in on-site holding lagoons. Afterward, the solids are separated for composting, and the water is recycled for future flush purposes [19]. In California, lagoon water may be used to irrigate crops, such as corn intended solely for silage, under strict regulation and as part of water conservation efforts [20]. However, frequent exposure of cows in free-stall pens to the recycled lagoon water may lead to the transmission and spread of drug-resistant bacteria from fecal sources between animals and the farm environment [21,22,23]. Excreted AMD can also continue to exert selection pressure for resistance in the manure, lagoon, and wastewater, depending on their rate of degradation, hydrophobicity, and sorption potential [24]. As a result, recycled lagoon water could contribute to the dissemination of AMR bacteria to other management units of a dairy that is exposed to such recycled lagoon water.

Recent surveillance studies on California dairies showed low AMR in indicator organisms (*E. coli* and *Enterococcus* spp.) from fecal samples of adult cows against drugs commonly administered to adult dairy cows, such as cephalosporins and penicillin, which suggests that these drugs are still effective in the treatment of dairy cows [25]. Unexpectedly, the same study detected higher rates of AMR to drugs approved for use only in non-lactating cattle, commonly youngstock, such as macrolides, florfenicol, and tiamulin. A large proportion of the study cows were healthy, and none had a record of treatments using macrolides, florfenicol, or tiamulin. However, study dairies used these drugs to treat the young stock, except for one dairy that raised the calves off-site from birth until just before their first calving [25]. Comparable levels of resistance were reported for *E. coli* isolates from Canadian cattle [26,27]. Such an observation could be explained by co-selection for resistance genes since florfenicol resistance was commonly observed in combination with resistance to tetracycline or sulfamethoxazole and is supported by the literature [28,29]. Alternatively, the presence of AMR, regardless of non-exposure to the same drugs in adult cows, may be explained by transmission of AMR bacteria from nulliparous heifers and the environment.

Previous studies have detected the presence of antimicrobial drug residues in lagoon water in dairies [20,30]. However, little is known about the associations between AMR of bacterial isolates originating from lagoon water and AMD residues in lagoon water. This study aimed to determine the role of recycled lagoon water in disseminating antibiotic-resistant bacteria and AMD residues on dairies. Our specific objectives were: (1) To determine the AMR phenotypes of indicator fecal bacteria (*E. coli* and *Enterococcus* spp. and *Streptococcus* spp.) isolated from lagoon water samples collected from nine dairies in California over two seasons, (2) to determine the presence of AMD (florfenicol, tilmicosin, penicillin, sulfamethoxazole and tetracycline) in lagoon samples, and (3) to investigate the association between AMD residues and AMR phenotypes of indicator bacteria isolated from lagoon samples.

## 2. Materials and Methods

### 2.1. Study Design and Sample Collection

The current study utilized a repository of lagoon and flush water samples from a total of 9 dairies across California that were enrolled in a previous longitudinal study [25]. Briefly, samples were collected from four dairies in Greater Southern California (GSCA), three dairies in Northern California (NCA), and two dairies in Northern San Joaquin Valley (NSJV). All the dairies raised calves onsite. The samples were collected from October 2018 to August 2019, spanning two seasons: the Fall/Winter season (October 2018 to March 2019) and the Spring/Summer season (March to August 2019). During each season, the dairies were visited five times, approximately every four to five weeks. Lagoon sampling was conducted as part of a follow-up study on a cohort of cows, with the first sampling point occurring at close-up (approximately 0–14 days pre-calving) and the remaining four sampling points at 30, 60, 90, and 120 days in milk post-calving. Samples were manually collected from the recycled lagoon water by scooping into 250 mL sterile sample bottles. However, if accessing lagoon water was difficult or dangerous, samples were collected from the recycled lagoon water during the flushing of a dairy pen instead. Collected samples were transported on dry ice to the DairyEpi Lab (UC Davis Veterinary Medicine Teaching and Research Center, Tulare, CA, USA) for same-day processing. Bacterial culture was performed on aliquots of the fresh samples, while the rest of the samples were stored at −80 °C for AMD residue analysis after the end of the sampling period. Overall, the study generated a repository of 10 lagoon water samples for each of the study dairies. The samples were collected every 4–5 weeks over a period of one year.

### 2.2. Bacteriological Isolation and Identification

The *E. coli* and Enterococcus ChromoSelect agar (Sigma-Aldrich, St. Louis, MO, USA) were used to identify and isolate *E. coli* and *Enterococcus* spp., respectively. However, Enterococcus ChromoSelect agar cannot distinguish between *Enterococcus* spp. and *Streptococcus* spp. based solely on colony characteristics. Previous confirmation using sequencing and MALDI-TOF of colonies initially identified as *Enterococcus* spp. on ChromoSelect agar showed that about 50% were actually *Enterococcus* spp., and the remaining 50% were *Streptococcus* spp. Based on this finding, these isolates were henceforth referred to collectively as *Enterococcus* spp. and *Streptococcus* spp. (ES) for simplicity. To isolate each bacterial type, the lagoon water samples were spread onto *E. coli* and Enterococcus ChromoSelect agar plates and incubated at 37 °C for 18–24 h. *E. coli* colonies were identified by their fluorescent blue color on *E. coli* ChromoSelect agar when viewed under UV light, while ES colonies were identified by their blue-green color on Enterococcus ChromoSelect agar. Two separate colonies from each sample were then purified on blood agar with 5% sheep blood and incubated at 37 °C for 24 h in preparation for antimicrobial susceptibility testing (AST).

### 2.3. Antimicrobial Susceptibility Testing

To test for antimicrobial susceptibility, the broth microdilution method was utilized. This involved determining the minimum inhibitory concentration (MIC) of the test drugs for the different isolates. The MIC value refers to the lowest concentration (in μg/mL) of an AMD that can inhibit bacterial growth. All *E. coli* and ES isolates were tested against a panel of drugs on BOPO7 MIC plates (Thermo Fisher, Waltham, MA, USA). The interpretation of MIC breakpoints followed the guidelines set by the Clinical and Laboratory Standards Institute (CLSI), when available for a specific drug-organism combination. For drug-organism combinations without CLSI interpretation, breakpoints from EUCAST (European Committee on Antimicrobial Susceptibility Testing) and other publications were used (Supplemental Tables S1 and S2). *E. coli* phenotypic susceptibility was determined for the drugs including ampicillin, ceftiofur, danofloxacin, enrofloxacin, florfenicol, gamithromycin, gentamicin, neomycin, spectinomycin, sulphadimethoxine, tetracycline, tiamulin, tilmicosin, tildipirosin, trimethoprim-sulfamethoxazole, tulathromycin, and tylosin. ES phenotypic susceptibility was determined for ampicillin, florfenicol, gamithromycin, penicillin, tetracycline, tiamulin, tilmicosin, tildipirosin, tulathromycin, tylosin. The isolates were categorized as susceptible or resistant based on the MIC breakpoints. Parallel interpretation was used on the results of AST at the sample level; if only one of the two isolates in a sample tested AMR-positive, we classified such a sample as resistant to that specific AMD.

### 2.4. ELISA Test for AMD Residues

To detect AMD residues in the samples, an ELISA kit (Creative Diagnostics, Shirley, NY, USA) was used to determine the presence or absence of a drug. The drugs tested and the ELISA kits used were as follows: florfenicol (cat# DEIA039), tilmicosin (cat# DEIA038), penicillin (DEIABL-QB25), tetracycline (cat# DEIA046), and sulfamethoxazole (cat#DEIABL-QB9). These ELISA kits were developed for quantitative measurements of antimicrobial drug residues in tissues or animal products and were adapted to lagoon water. Briefly, 20 μL of each lagoon sample was transferred into a micro-centrifuge tube containing 380 μL of extraction buffer and vortexed for 1 min to mix thoroughly. Each sample was then aliquoted into a 96-well plate and stored at −20 °C until ready for testing by ELISA. All samples, including standards and spike controls, were assayed in duplicates (50 μL/well). Each AMD standard and spike control was included in the test kits. The manufacturer-recommended cut-offs used to categorize the ELISA results were as follows: florfenicol 0.05 ppb, tilmicosin 0.5 ppb, penicillin 0.1 ppb, tetracycline 0.2 ppb, and sulfamethoxazone 0.5 ppb. Samples with estimated residue concentrations greater than or equal to the cut-off value were classified as positive for the specific drug, and duplicate reactions for each sample were interpreted in parallel.

### 2.5. Statistical Analysis

The percentage of lagoon samples with AMD residues and the percentage of isolates with AMR against each AMD were estimated using robust estimation methods and their standard errors and associated 95% CI reported across seasons, regions, and repeated sampling points. To investigate the association between AMD residues in lagoon samples and AMR phenotypes of bacteria isolated from lagoon samples, logistic regression models were specified. In addition to AMD residues, other predictors considered included region, season, and sampling point. To account for lack of independence in observations within each study farm, robust standard error estimates were calculated over the 9 study clusters (farms). After univariate models were specified, a manual forward model-building procedure was employed, starting with significant predictors while always forcing the exposure of interest (drug residues) that resulted in the best-fitting model. The drug residue resulting in the best-fitting model was determined using the lowest Akaike information criterion (AIC). Each model was then adjusted for confounding by season and/or region based using the change-in-estimate criterion, with covariates retained if their inclusion altered the effect estimate by 20% or greater. Finally, the model was tested for two-way interactions between the drug residue and each of the seasons and regions. The Hosmer and Lemeshow tests were used to assess the goodness of fit of the final models after variable selection, confounding assessment, and testing for interaction. A 5% level of significance was observed for all comparisons using Stata version 17 (Stata Corp. LLC, College Station, TX, USA). 

## 3. Results

This study included nine California dairies, each visited 10 times at 4- to 5-week intervals to collect lagoon samples. Due to access issues, a total of 89 lagoon samples were collected over 90 visits—Herd 7 missed one sampling point. From each lagoon sample, two *E. coli* colonies and two ES colonies were selected after culture and identification. In total, 150 *E. coli* and 163 ES isolates from these samples were tested for antimicrobial susceptibility. To assess antimicrobial resistance (AMR) at the lagoon sample level for each bacterial species, parallel interpretation was used, resulting in 75 *E. coli* and 82 ES interpretation pairs for analysis. Among the nine dairies, only Herd 2 had a complete set of results, with all 10 lagoon samples for both *E. coli* and ES having their AMR status determined (Table 1).

### 3.1. Prevalence of Antimicrobial Resistance

The prevalence of AMR in *E. coli* isolates from lagoon samples is summarized in Table 2. In this context, a lagoon sample is considered resistant to a specific drug if at least one isolate type from the sample is resistant to that drug. All lagoon samples (100% ± 0.0) showed resistance to tiamulin, tilmicosin, or tylosin. A high prevalence of resistance was also observed against florfenicol (96.0% ± 2.0) and gamithromycin (92.0% ± 1.9). Conversely, resistance to other AMDs was low, with less than 10% of the samples exhibiting resistance. These included ceftiofur, danofloxacin, enrofloxacin, gentamicin, neomycin, spectinomycin, trimethoprim-sulfamethoxazole, and tulathromycin.

Among the ES isolates, resistance was highly common against several macrolides and related drugs (Table 3). Nearly all isolates were resistant to tildipirosin (97.6% ± 1.7) and tilmicosin (98.8% ± 1.2), while florfenicol resistance was also widespread (95.1% ± 2.0). Moderate levels of resistance were observed for gamithromycin (51.2% ± 5.2), tetracycline (50.0% ± 6.3), tulathromycin (45.1% ± 5.1), and tylosin (30.5% ± 6.9). In contrast, relatively few isolates were resistant to penicillin (8.5% ± 2.4) or ampicillin (4.9% ± 1.9).

**Figure 1:**
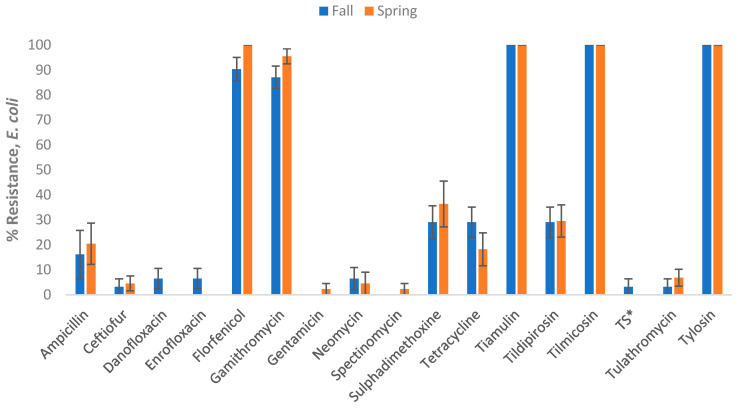
Percentages of lagoon samples with antimicrobial-resistant *Escherichia coli* categorized by season (n = 75). Lagoon samples were collected from 9 California dairies at 4- to 5-week intervals during the Fall/Winter (Fall) and Winter/Spring (Spring) seasons. A total of 10 samples were collected per dairy over the I year study period. Error bars represent one standard error of the mean. * TS refers to Trimethoprim Sulfamethoxazole.

**Figure 2:**
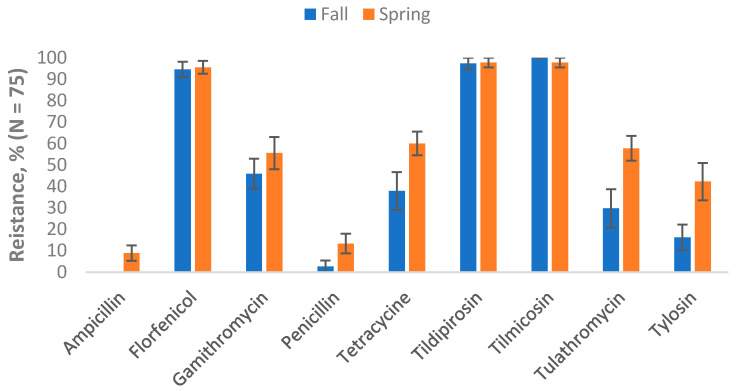
Percentages of lagoon samples with antimicrobial-resistant *Enterococcus* spp./*Streptococcus* spp. categorized by season (n = 82). Lagoon samples were collected from 9 California dairies at 4- to 5-week intervals during the Fall/Winter (Fall) and Winter/Spring (Spring) seasons. A total of 10 samples were collected per dairy over the one-year study period. Error bars represent one standard error of the mean.

Seasonality: Considering seasonality, the results showed that prevalence estimates of AMR in the study lagoon samples varied by season. Among the *E. coli,* isolates resistant to danofloxacin, enrofloxacin, or trimethoprim-sulfamethoxazole, all were collected in Fall/Winter. Conversely, *E. coli* isolates resistant to gentamicin or spectinomycin were all from samples collected in Spring/Summer. Higher resistance to ampicillin, ceftiofur, florfenicol, gamithromycin, sulphadimethoxine and tulathromycin was observed in the Spring compared to summer (Figure 1). Among the ES category, all the ampicillin-resistant isolates came from samples collected in Spring/Summer. Resistance to florfenicol, tilmicosin, and tildipirosin was similar across the two seasons, while relatively higher resistance in the Spring compared to Summer was observed for gamithromycin, penicillin, tetracycline, tulathromycin, and tylosin (Figure 2).

**Figure 3:**
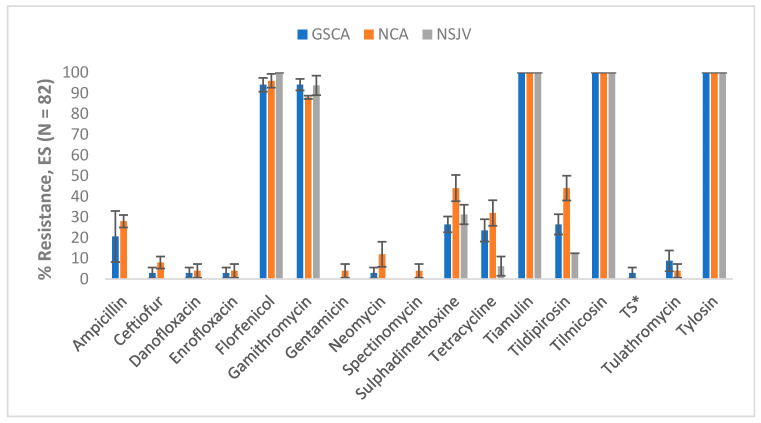
Percentages of antimicrobial-resistant *Escherichia coli* in lagoon water samples by region and drug (n = 75). Lagoon samples were collected from 9 California dairies at 4- to 5-week intervals (10 times per dairy). Error bars represent one standard error of the mean. * TS refers to Trimethoprim Sulfamethoxazole. GSCA: Greater Southern California; NCA: Northern California; NSJV: Northern San Joaquin Valley.

Regional Variations: Differences in antimicrobial resistance patterns were observed among *E. coli* isolates, although some were limited to specific drugs and regions (Figure 3). Resistance to ampicillin, ceftiofur, danofloxacin, enrofloxacin, gentamicin, neomycin, spectinomycin, trimethoprim-sulfamethoxazole, or tulathromycin was observed only in isolates from GSCA or NCA, but not in those from NSJV. For example, ampicillin resistance was recorded in 20.6% of samples from GSCA and 28.0% from NCA, while none of the samples from NSJV showed resistance. Similarly, resistance to ceftiofur, fluoroquinolones (danofloxacin and enrofloxacin), and trimethoprim-sulfamethoxazole was low but confined to GSCA or NCA. Tetracycline and sulphadimethoxine resistance were highest in NCA (32.0% and 44.0%, respectively), followed by GSCA (23.5% and 26.5%), and lowest in NSJV (6.3% and 31.3%). In contrast, florfenicol resistance was consistently high across all regions (94–100%), and tiamulin, tilmicosin, and tylosin resistance was universal in all samples, regardless of region.

**Figure 4:**
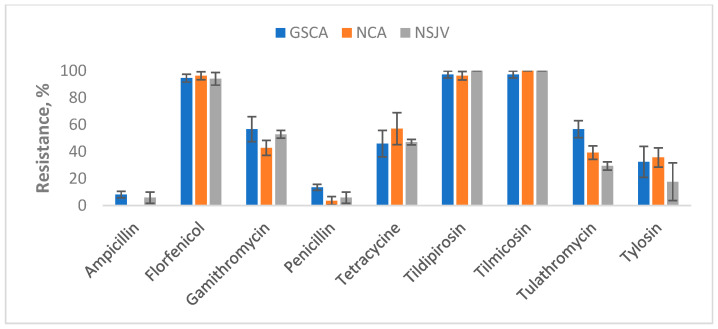
Percentages of antimicrobial-resistant *Enterococcus* spp./*Streptococcus* spp. lagoon water samples by region and AMD resistance (n = 82). Lagoon samples were collected from 9 California dairies at 4- to 5-week intervals (10 times per dairy). Error bars represent one standard error of the mean. GSCA: Greater Southern California; NCA: Northern California; NSJV: Northern San Joaquin Valley.

For ES isolates, regional patterns showed similar trends of high resistance to macrolides and florfenicol, with minor differences among regions (Figure 4). Ampicillin resistance was detected only in GSCA (8.1%) and NSJV (5.9%), but not in NCA samples. Penicillin resistance followed a similar pattern, being highest in GSCA (13.5%), but rare in NCA (3.6%) and NSJV (5.9%). Moderate levels of tetracycline resistance were observed across regions, ranging from 45.9% in GSCA to 57.1% in NCA. Resistance to tulathromycin was highest in GSCA (56.8%), followed by NCA (39.3%), and lowest in NSJV (29.4%). Gamithromycin resistance varied between 42.9% and 56.8% across regions, without a clear trend. In contrast, florfenicol resistance remained uniformly high (>94%) across all three regions, and complete resistance to tildipirosin and tilmicosin was observed in NSJV and NCA, and nearly complete resistance also in GSCA.

### 3.2. Estimates of Antimicrobial Drug Residues

The results of drug residue estimation in lagoon samples using ELISA are summarized in Table 4. The overall prevalence estimates for florfenicol and tilmicosin residues in lagoon samples were greater than 90%. The remaining three drugs were observed at lower prevalences, with the lowest observed drug residue being penicillin (3.4% ± 1.9).

**Figure 5:**
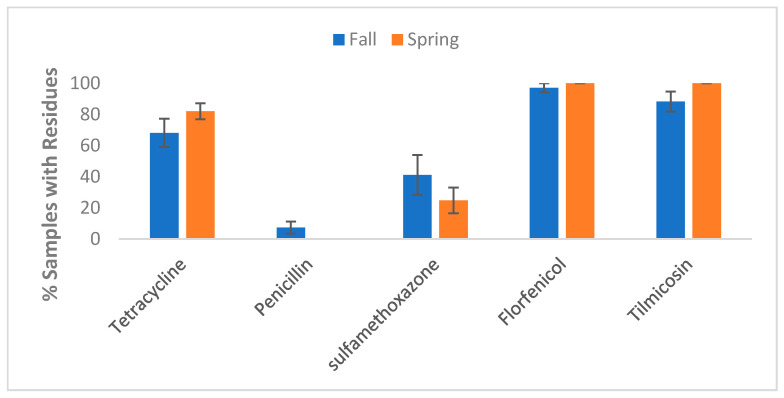
Prevalence of AMD residue in lagoon samples by drug and season. Lagoon samples were collected from 9 California dairies at 4- to 5-week intervals (10 times per dairy). Error bars represent one standard error of the mean. Fall (n = 68); Spring (n = 89).

Seasonality: Seasonal differences were observed in the presence of AMD residues in lagoon water samples (Figure 5). Compared with Fall/Winter, a greater proportion of samples collected in the Spring/Summer contained florfenicol (100% vs. 97.1%), tilmicosin (100% vs. 88.2%), or tetracycline residues (82.0% vs. 68.1%). In comparison, fewer samples had sulfamethoxazone residues (24.7% vs. 41.2%), and none had penicillin residues.

**Figure 6:**
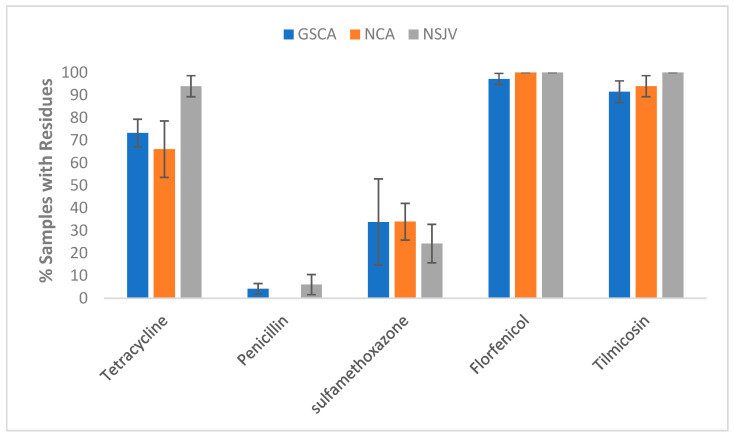
Prevalence of AMD residue in lagoon samples by drug and by region (Northern California, NCA; Northern San Joaquin Valley, NSJV; Greater Southern California, GSCA). Lagoon samples were collected from 9 California dairies at 4- to 5-week intervals (10 times per dairy). Error bars represent one standard of the mean. GSCA: Greater Southern California (n = 71); NCA: Northern California (n = 53); NSJV: Northern San Joaquin Valley (n = 33).

Regional variations: Regional comparisons also revealed differences in the distribution of residues (Figure 6). Tetracycline residues were most frequently detected in NSJV samples (93.9%), compared with GSCA (73.2%) and NCA (66.0%). Penicillin residues were rare overall but detected in GSCA (4.2%) and NSJV (6.1%) samples, with none found in NCA. Sulfamethoxazole residues showed comparable prevalence across GSCA (33.8%) and NCA (34.0%) but were somewhat lower in NSJV (24.2%). Florfenicol residues were nearly ubiquitous across all regions (97.2–100%), as were tilmicosin residues (91.6–100%).

**Figure 7:**
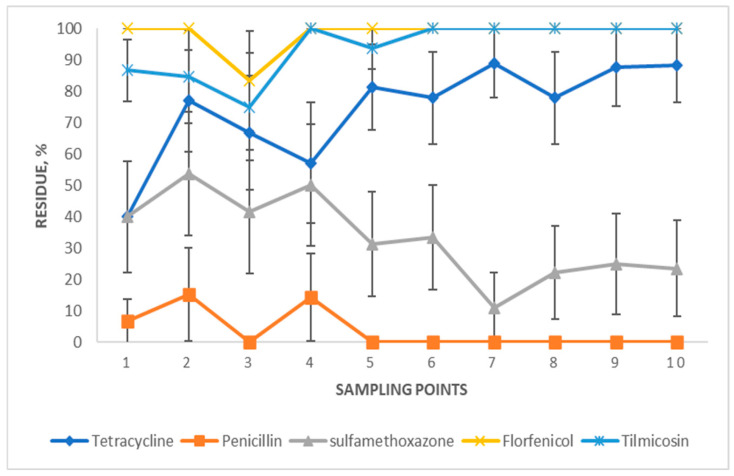
Percentages of lagoon samples with AMD residue by sampling point. Lagoon samples were collected from 9 California dairies at 4- to 5-week intervals (10 times per dairy). Error bars represent one standard error of the mean.

Variations across sampling time points: The variations in proportions of samples with AMD residues across the 10 different sampling points over two seasons are depicted in Figure 7. Although not statistically significant, an increasing trend was observed in tilmicosin or tetracycline residues from sampling points 1 to 10, while a decreasing trend was observed in penicillin or sulfamethoxazone residues during the same period. Florfenicol was detected at 100% at all sampling points except point 3, which had a slight deduction to 85%.

### 3.3. Logistic Regression Models for AMR

The results of the final logistic regression models for *E. coli* isolates are summarized in Table 5. *E. coli* isolates were 100% resistant to tiamulin, tilmicosin or tylosin; hence, models could not be specified. The odds for both danofloxacin and enrofloxacin resistance in *E. coli* isolates in the presence of tilmicosin residues were 0.04 times the odds of those without tilmicosin residues (*p*-value < 0.05). When contrasting the presence of drug residues among *E. coli* isolates in NSJV, *E. coli* isolates from lagoon samples with sulfamethoxazone residues had almost 7 times higher odds of sulphadimethoxazone resistance (OR = 6.7, 95% CI = 0.1–13.2, *p* < 0.05) than those from samples without sulfamethoxazone residues. The odds of tildipirosin resistance in *E. coli* isolates in the presence of tilmicosin residues were 0.07 times the odds of those without tilmicosin residues (*p*-value < 0.05). The odds of tildipirosin resistance in *E. coli* isolates from the regions NCA and NSJV were 2.9 and 0.4 times the odds of reference region (GSCA) (*p*-value < 0.05)

The results of the logistic regression model for ES isolates are summarized in Table 6. The odds of tetracycline resistance in ES isolates in the presence of sulfamethoxazone residues were 4.11 times the odds of those without sulfamethoxazone residues (*p*-value < 0.05). The odds of tetracycline resistance in ES isolates in Spring/Summer were 5.2 times the odds of those in Fall/Winter (*p*-value < 0.05). In Spring/Summer, ES isolates with the presence of sulfamethoxazone residues had over 4 times higher odds of tetracycline resistance compared with ES isolates without the presence of sulfamethoxazone residues (OR = 4.1, 95% CI = 0.7–7.5, *p* = 0.02). The odds of tulathromycin resistance in ES isolates in Spring/Summer or in NSJV were 3.1 times and 0.3 times the odds of those in Fall/Winter or in GSCA, respectively (*p*-value < 0.05). The odds of tylosin resistance in ES isolates in the presence in Spring/Summer were 4.7 times the odds of those in Fall/Winter (*p*-value < 0.05).

Table 7 summarizes the estimated odds ratios (OR) for the joint effects of drug residues and geographic region or season on antimicrobial resistance in *E. coli* and *Enterococcus* spp. For *E. coli*, the presence of sulfamethoxazole residues was significantly associated with higher odds of sulfadimethoxine resistance in certain regions. Compared with the GSCA referent, isolates from the NCA region with sulfamethoxazole residues had five times the odds of resistance (OR = 5.00; 95% CI: 1.27–19.75; *p* = 0.02). Similarly, in the NSJV region, the odds of resistance were elevated (OR = 6.00; 95% CI: 0.91–39.46; *p* = 0.06) compared with GSCA, although this result was not statistically significant. When comparing NCA to NSJV, the odds of resistance were also elevated (OR = 4.13; 95% CI: 0.44–38.29; *p* = 0.21), but not statistically significant. Notably, in the absence of sulfamethoxazole residues, the presence of these residues in the NSJV region was associated with a significantly higher odds of resistance (OR = 6.67; 95% CI: 2.50–17.76; *p* < 0.01), whereas differences for GSCA and NCA were not statistically significant. For Enterococcus spp., seasonality appeared to modify the relationship between sulfamethoxazole residues and tetracycline resistance. Compared with fall samples without sulfamethoxazole residues, fall samples with sulfamethoxazole residues had significantly higher odds of tetracycline resistance (OR = 4.11; 95% CI: 1.70–9.44; *p* < 0.01). No significant associations were observed in spring samples, regardless of sulfamethoxazole residue status.

## 4. Discussion

Our analysis of antimicrobial residues showed that over 90% of the lagoon samples contained residues of florfenicol and tilmicosin. In contrast, the other three drugs had much lower detection rates, with penicillin being the least common at 3.4% ± 1.9. This variation in the prevalence of drug residues highlights the need for further investigation into their pathways and implications for the ecosystem. The observed low level of penicillin residues could be attributed to low usage in dairy cattle due to its long withdrawal period for milk and meat [31]. In contrast, a relatively high percentage of lagoon samples had florfenicol or tilmicosin residues. In the US, florfenicol is not labeled for use in lactating dairy cows, but it is labeled for use in calves [32]. Since the samples were collected from recycled lagoon water used to flush excreta across different management units of a dairy, including free stalls, dry lots, exercise yards, hospital barns, and calf and heifer housing areas, the florfenicol residue could be a result of use in calves and heifers. Additionally, drugs excreted in the feces and urine of treated cattle, along with antimicrobial-resistant bacteria in the environment, may contaminate the lagoon water, potentially explaining the high percentage of *E. coli* and ES isolates that were resistant to florfenicol. An alternative explanation for the observed resistance pattern could be co-selection for linked resistance genes [33]. The resistance genes for florfenicol and tilmicosin are linked and are inherited as a block [28,34], which may explain the relatively high resistance of both *E. coli* and ES isolates against these drugs. Moreso, nearly 100% of *E. coli* and ES isolates showed resistance against tiamulin. Although *E. faecalis* is intrinsically resistant to lincosamides, pleuromutilins, and streptogramin A by production of the ABC transporter Lsa(A), *E. faecium* is naturally susceptible to pleuromutilins. As tiamulin is one of the pleuromutilins derivatives, it is possible that most of the study isolates were *E. faecalis*.

Numerical differences in the percentage of isolates resistant to drugs between seasons, regions, and sampling points were detected but not statistically significant. The differences between the Fall/Winter and Spring/Summer seasons could possibly be attributed to the impact of the climate, seasonal temperature variations, management practices, and seasonality of some diseases. Similarly, the variations among regions and sampling points may result from variations in environmental factors, disease prevalence, and the use of AMD in cows to treat and prevent diseases.

The logistic regression models identified several significant associations between different predictors and AMR outcomes. Among the significant associations, the presence of tilmicosin residues was associated with a decrease in the frequency of resistance against danofloxacin or enrofloxacin in *E. coli* isolates. The observation could be explained by the phenomenon of collateral sensitivity, where the presence of one drug decreases resistance to another drug [35]. Other associations observed included the interaction between sulfamethoxazone residues and Region NSJV, which was associated with higher odds of resistance in sulphadimethoxine in *E. coli* isolates. The presence of tilmicosin residues and Region NSJV was linked to lower odds of resistance in *E. coli* isolates against tildipirosin, while Region NCA was related to higher odds of resistance in *E. coli* isolates against tildipirosin. These associations have not been previously reported and should be explored more. We noticed that there were several significant negative associations between AMD residues and AMR observed in *E. coli* isolates, such as tilmicosin residues and resistance against danofloxacin or enrofloxacin, sulfamethoxazone residues and resistance against sulphadimethoxine, and tilmicosin residues and resistance against tildipirosin. However, such negative associations seem unconvincing, though reported in a previous study [36]. This may imply that the resistance determinants are incompatible with one another, or that such negative associations potentially indicate unidentified risk factors [37,38]. Several associations could not be tested due to the complete correlation between drug residues and resistance or susceptibility (Tables S3 and S4). Such complete correlations between an exposure (drug residues) and an outcome (resistance) result in a lack of variance, which precludes models from converging.

Our analysis also revealed that not all AMD residues were associated with AMR, which may further underscore the complexity of AMR and suggest other potential mechanisms for its development in the dairy environment. A specific AMD residue may not directly influence AMR against the corresponding drug. For example, in the logistic regression models for ES isolates, tetracycline residues rather than florfenicol residues were selected for the final model, the outcome of which was resistance against florfenicol. Similarly, sulfamethoxazone residues rather than tetracycline residues were selected for the final model of which the outcome was resistance against tetracycline. These observations could similarly be explained by the phenomenon of cross-resistance [35]. It was previously reported that antimicrobial drug use on livestock farms can affect the abundance of antimicrobial resistance genes in either the corresponding or other antimicrobial classes [39,40]. Further research is needed to explore the correlation between AMD residues and AMR against the corresponding or non-corresponding drug in samples isolated from livestock environments.

Misclassification bias was a potential source of bias when measuring the presence of AMD residues and AMR phenotypes of the indicator bacteria isolated from the lagoon samples. The presence and absence of drug residues were classified based on the cut-off values and sensitivity of the ELISA kits. Here, imperfect sensitivity and specificity result in false-negative and false-positive results, respectively, which may cause misclassification bias. The AMR phenotypes were identified by the breakpoint values of MIC, which may also result in misclassification bias.

This study was limited by the relatively small sample size, with only 75 *E. coli* and 82 ES isolates out of 89 lagoon samples, which reduced the statistical power and precluded reliable regional comparisons. Observed differences likely reflect farm-specific rather than regional variation, and the absence of detailed AMD usage data, animal source information, and lagoon design parameters restricted interpretation of seasonal or management influences on AMR. Moreover, only 5 drugs were tested using ELISA, which, while practical, is less sensitive than LC–MS/MS and may underestimate residue levels. Testing for additional drug residues could have allowed comparison with bacterial resistance across more drug classes. In addition, the rate of degradation of said drugs in the lagoon environment is not known; hence, residues could have been missed. Finally, this study could have included the assessment of AMR genes as outcomes, which could further supplement the associations observed with AMR phenotypes.

## 5. Conclusions

The lagoon samples from 9 California dairies showed extremely high levels of florfenicol or tilmicosin residues, high levels of tetracycline residues, but considerably low levels of penicillin residues. The proportion of AMR *E. coli* and ES isolates from lagoon water samples varied by season, region, and sampling point. The regression model results showed that the presence of tilmicosin residues was associated with a decrease in the frequency of resistance against danofloxacin or enrofloxacin in *E. coli.* Due to the complete correlation between residues and resistance or susceptibility, some associations could not be modeled. Among *E. coli* isolates, complete correlations were observed between various residues and resistance, such as penicillin residues with resistance to multiple AMD. For ES isolates, similar complete correlations were identified, highlighting the need for further research to confirm these associations. The findings of the current study can inform future research and stewardship practices related to manure management on dairies.

## Figures and Tables

**Table 1:** List of the dairy herd IDs and the number of lagoon samples collected from each dairy. From each sample, two isolates of *E. coli* and *Enterococcus* spp./*Streptococcus* spp. (ES) strains were obtained. Lagoon samples were collected every four weeks from nine California dairies over two seasons, spanning 2018 to 2019.

Herd ID	Organism Pairs		
	*E. coli*	ES	Total
1	9	9	18
2	10	10	20
4	6	8	14
5	9	10	19
6	8	9	17
7	8	8	16
8	9	9	18
9	9	10	19
10	7	9	16
Total	75	82	157

**Table 2:** Percentages of antimicrobial-resistant *E. coli* (n = 75) from lagoon water samples. Lagoon samples were collected from 9 California dairies at 4- to 5-week intervals (10 times/dairy). Error bars represent one standard error of the mean.

*E. coli* (n = 75)				
Antimicrobial Agent	MIC Breakpoints (μg/mL)	% Resistant	SE	95% CI
Ampicillin	≥16	18.67	6.73	7.63, 38.95
Ceftiofur	≥4	4.00	1.93	1.29,11.72
Danofloxacin	≥0.5	2.67	1.73	0.59, 11.28
Enrofloxacin	≥0.5	2.67	1.73	0.59, 11.28
Florfenicol	≥4	96.00	2.01	87.77, 98.77
Gamithromycin	≥8	92.00	1.87	86.47, 95.39
Gentamicin	≥8	1.33	1.32	0.13, 12.05
Neomycin	≥8	5.33	2.89	1.48, 17.44
Spectinomycin	≥64	1.33	1.32	0.13, 12.05
Sulphadimethoxine	>256	33.33	4.11	24.61, 43.37
Tetracycline	≥4	22.67	4.67	13.69, 35.14
Tiamulin	≥32	100.00	0.00	
Tildipirosin	≥8	29.33	5.05	19.14, 42.13
Tilmicosin	≥16	100.00	0.00	
Trimethoprim-sulfamethoxazole	>2/38	1.33	1.32	0.13, 12.05
Tulathromycin	≥32	5.33	2.82	1.52, 16.95
Tylosin	≥16	100.00	0.00	

**Table 3:** Percent of antimicrobial resistance in *Enterococcus* spp. and *Streptococcus* spp. isolated from lagoon samples of 9 California dairies from 2018 to 2019.

ES (N = 82)				
Antimicrobial Agent	MIC Breakpoints (μg/mL)	% Resistant	SE	95% CI
Ampicillin	≥16	4.88	1.91	1.95, 11.70
Florfenicol	≥4	95.12	1.95	88.08, 98.09
Gamithromycin	≥8	51.22	5.15	39.49, 62.82
Penicillin	≥8	8.54	2.38	4.42, 15.86
Tetracycline	≥4	50.00	6.34	35.79, 64.21
Tildipirosin	≥8	97.56	1.65	89.04, 99.50
Tilmicosin	≥16	98.78	1.24	88.33, 99.88
Tulathromycin	≥32	45.12	5.09	33.86, 56.91
Tylosin	≥16	30.49	6.90	17.15, 48.18

**Table 4:** Prevalence of AMD residues in lagoon samples by drug and herd. Lagoon samples were collected from 9 California dairies at 4- to 5-week intervals (10 times per dairy).

Herd	Region	Number of Samples	% Samples Positive for Drug Residue (SE)				
			Tetracycline	Penicillin	Sulfamethoxazone	Florfenicol	Tilmicosin
1	GSCA	10	70.00 (14.49)	10.00 (9.49)	90.00 (9.49)	100.00 (0.00)	70.00 (14.49)
2	GSCA	10	60.00 (15.49)	10.00 (9.49)	10.00 (9.49)	90.00 (9.49)	90.00 (9.49)
4	GSCA	10	70.00 (14.49)	0.00	30.00 (14.49)	100.00 (0.00)	100.00 (0.00)
5	GSCA	10	90.00 (9.49)	0.00	0.00	100.00 (0.00)	100.00 (0.00)
6	NSJV	10	100.00 (0.00)	10.00 (9.49)	40.00 (15.49)	100.00 (0.00)	100.00 (0.00)
7	NSJV	9	88.89 (10.48)	0.00	11.11 (10.48)	100.00 (0.00)	77.78 (13.86)
8	NCA	10	90.00 (9.49)	0.00	20.00 (12.65)	100.00 (0.00)	100.00 (0.00)
9	NCA	10	50.00 (15.81)	0.00	50.00 (15.81)	100.00 (0.00)	100.00 (0.00)
10	NCA	10	50.00 (15.81)	0.00	40.00 (15.49)	100.00 (0.00)	100.00 (0.00)
Total		89	74.16 (4.64)	3.37 (1.91)	32.58 (4.97)	98.88 (1.12)	93.26 (2.66)

SE: Standard error; GSCA: Greater Southern California; NCA: Northern California; NSJV: Northern San Joaquin Valley. No lagoon samples were collected from herd 3.

**Table 5:** Final logistic regression models for the association between drug residues and AMR in *Escherichia coli* isolated from lagoon samples of 9 California dairies from 2018 to 2019 (n = 75 samples).

Outcome ^1^	Predictor ^2^	Coefficient	Robust SE	*p* Value	OR	95% CI	
						Lower	Upper
Ampicillin	Sulfamethoxazone	1.33	0.77	0.08	3.79	0.84	17.18
	Region (NCA)	0.49	0.62	0.43	1.63	0.49	5.48
Ceftiofur	Tetracycline	−0.20	1.73	0.89	0.82	0.06	12.10
	Season (Spring)	0.41	1.60	0.80	1.50	0.07	34.25
	Region (NCA)	1.03	1.04	0.32	2.81	0.36	21.75
Danofloxacin	Tilmicosin	−0.15	1.31	0.02	0.04	<0.01	0.56
Enrofloxacin	Tilmicosin	−0.15	1.31	0.02	0.04	<0.01	0.56
Florfenicol	Penicillin	−3.43	1.87	0.07	0.03	<0.01	1.26
	Region (NCA)	−0.26	1.37	0.85	0.77	0.05	11.35
Gamithromycin	Penicillin	−1.94	1.86	0.30	0.14	<0.01	5.53
	Season (Spring)	0.93	0.95	0.33	2.53	0.39	16.32
	Region (NCA)	−1.15	0.64	0.07	0.32	0.09	1.11
	Region (NSJV)	−0.10	0.75	0.89	0.90	0.21	3.93
Neomycin	Sulfamethoxazone	0.80	1.52	0.60	2.23	0.11	44.16
	Region (NCA)	1.55	0.89	0.08	4.70	0.82	26.93
Sulphadimethoxine	Sulfamethoxazone	−0.12	0.68	0.86	0.89	0.23	3.38
	Region (NCA)	0.37	0.55	0.50	1.45	0.50	4.26
	Region (NSJV)	−0.22	0.31	0.48	0.80	0.43	1.48
	Sulfamethoxazone × Region (NCA)	1.32	1.04	0.24	3.44	0.45	26.48
	Sulfamethoxazone × Region (NSJV)	2.01	0.85	0.02	7.50	1.43	39.34
Tetracycline	Sulfamethoxazone	0.03	0.65	0.96	1.03	0.29	3.67
	Season (Spring)	−0.61	0.54	0.26	0.54	0.19	1.57
	Region (NCA)	0.46	0.40	0.25	1.58	0.72	3.47
	Region (NSJV)	−1.50	0.85	0.08	0.22	0.04	1.19
Tildipirosin	Tilmicosin	−2.69	1.12	0.02	0.07	0.01	0.61
	Region (NCA)	1.07	0.37	<0.01	2.90	1.41	5.98
	Region (NSJV)	−0.97	0.47	0.04	0.38	0.15	0.96
Tulathromycin	Sulfamethoxazone	−0.43	0.91	0.64	0.65	0.11	3.86
	Season (Spring)	0.85	0.91	0.35	2.35	0.40	13.90
	Region (NCA)	−0.93	1.06	0.38	0.39	0.05	3.16

GSCA: Greater Southern California; NCA: Northern California; NSJV: Northern San Joaquin Valley. ^1^ Specific drug residues were perfectly correlated with susceptibility or resistance. Penicillin residues, tetracycline residues, sulfamethoxazone residues, florfenicol residues or tilmicosin residues were all completely correlated with resistance or susceptibility of *E. coli* against gentamycin, spectinomycin or trimethoprim-sulfamethoxazole. The outcomes of AMR in *E. coli* were 100% resistant to tiamulin, tilmicosin or tylosin; hence, models could not be specified. For a complete list of variables correlated with resistance or susceptibility, see Supplementary Table S1. ^2^ Best fitting models, using the lowest Akaike Information Criterion estimate, were identified after offering each of the predictor variables for penicillin, tetracycline, sulfamethoxazole, florfenicol, or tilmicosin residues. Models were adjusted for confounders and effect-modification between the residues resulting in the best fitting model, by season and region. Season Fall and Region GSCA were used as references.

**Table 6:** Final logistic regression models for the association between drug residues and AMR in *Enterococcus* spp./*Streptococcus* spp. isolated from lagoon samples of 9 California dairies from 2018 to 2019 (n = 82 samples).

Outcome ^1^	Predictor ^2^	Coefficient	Robust SE	*p* Value	OR	95% CI	
						Lower	Upper
Ampicillin	Sulfamethoxazone	0.84	1.05	0.43	2.31	0.29	18.41
	Region (NSJV)	−0.32	0.83	0.70	0.73	0.14	3.71
Florfenicol	Tetracycline	0.03	1.13	0.98	1.04	0.11	9.43
Gamithromycin	Tilmicosin	−1.40	1.03	0.17	0.25	0.03	1.86
	Season (Spring)	0.53	0.46	0.25	1.70	0.69	4.14
Penicillin	Sulfamethoxazone	0.83	1.11	0.46	2.30	0.26	20.31
	Season (Spring)	1.90	1.10	0.09	6.69	0.77	58.35
Tetracycline	Sulfamethoxazone	1.41	0.42	<0.01	4.11	1.79	9.44
	Season (Spring)	1.64	0.34	<0.01	5.17	2.67	10.00
	Sulfamethoxazone × Season (Spring)	−1.71	0.80	0.03	0.18	0.04	0.87
Tildipirosin	Sulfamethoxazone	−0.73	1.65	0.66	0.48	0.02	12.31
Tulathromycin	Tetracycline	0.48	0.58	0.41	1.62	0.52	5.05
	Season (Spring)	1.14	0.48	0.02	3.12	1.12	8.03
	Region (NCA)	−0.69	0.44	0.12	0.50	0.21	1.19
	Region (NSJV)	−1.28	0.34	<0.01	0.28	0.14	0.54
Tylosin	Sulfamethoxazone	0.84	0.62	0.17	2.32	0.69	7.81
	Season (Spring)	1.54	0.41	<0.01	4.67	2.08	10.47

^1^ Specific drug residues were perfectly correlated with susceptibility or resistance. Penicillin residues, tetracycline residues, sulfamethoxazone residues, florfenicol residues or tilmicosin residues were all completely correlated with resistance or susceptibility of ES against tilmicosin. For a complete list of variables correlated with resistance or susceptibility, see Supplementary Table S2. ^2^ Best fitting models, using the lowest Akaike Information Criterion estimate, were identified after offering each of the predictor variables for penicillin, tetracycline, sulfamethoxazole, florfenicol, or tilmicosin residues. Models were adjusted for confounders and effect-modification between residues, resulting in the best-fitting model by season and region. Season Fall and Region GSCA were used as references.

**Table 7:** Estimated odds ratios (OR) for joint effects drug residues and Region/Season in organism isolated from lagoon samples of California dairies from 2018 to 2019.

Species	Outcome	Interaction	OR	Robust SE	*p* Value	95% CI	
						Lower	Upper
*E. coli*	Sulphadimethoxine	Sulfamethoxazone ×Region GSCA	Referent				
		Sulfamethoxazone × Region NCA	5.00	3.50	0.02	1.27	19.75
		Sulfamethoxazone × Region GSCA	Referent				
		Sulfamethoxazone × Region NSJV	6.00	5.77	0.06	0.91	39.46
		Sulfamethoxazone × Region NCA	Referent				
		Sulfamethoxazone × Region NSJV	4.13	4.70	0.21	0.44	38.29
		No Sulfamethoxazone × Region GSCA	Referent				
		Sulfamethoxazone × Region GSCA	0.88	0.61	0.86	0.23	3.38
		No Sulfamethoxazone × Region NCA	Referent				
		Sulfamethoxazone × Region NCA	3.06	2.41	0.16	0.65	14.30
		No Sulfamethoxazone × Region NSJV	Referent				
		Sulfamethoxazone × Region NSJV	6.67	3.33	<0.01	2.50	17.76
ES	Tetracycine	Sulfamethoxazone × Season Fall	Referent				
		Sulfamethoxazone × Season Spring	0.93	0.63	0.92	0.25	3.55
		No Sulfamethoxazone × Season Fall	Referent				
		Sulfamethoxazone × Season Fall	4.11	1.74	<0.01	1.70	9.44
		No Sulfamethoxazone × Season Spring	Referent				
		Sulfamethoxazone × Season Spring	0.74	0.52	0.67	0.19	2.91

GSCA: Greater Southern California; NCA: Northern California; NSJV: Northern San Joaquin Valley.

## Data Availability

The data presented in this study are available on request from the corresponding author. The data are not publicly available due to privacy restrictions.

## Supplementary Materials

The following supporting information can be downloaded at: https://www.mdpi.com/article/10.3390/vetsci12100960/s1. Table S1: Percent of antimicrobial resistance in *Escherichia coli* isolated from lagoon samples of 9 California dairies from 2018 to 2019. Table S2: Percent of antimicrobial resistance in *Enterococcus spp.* and *Streptococcus spp.* isolated from lagoon samples of 9 California dairies from 2018 to 2019. Table S3: Final logistic regression models for the association between drug residue and AMR in *Escherichia coli* isolated from lagoon samples of 9 California dairies from 2018 to 2019 (n = 75 samples). Table S4: Final logistic regression models for the association between drug residue and AMR in *Enterococcus spp./Streptococcus spp.* isolated from lagoon samples of 9 California dairies from 2018 to 2019 (n = 82 samples).
