# Supplementary material for: Analysis of Antimicrobial Residues and Resistance Profiles of Escherichia coli and Enterococcus spp. in Lagoon Water from California Dairies
Source: Vet Sci. 2025 Oct 8;12(10):960. doi: 10.3390/vetsci12100960 (PMC12568054; doi:10.3390/vetsci12100960)
Supplement: Supplementary file 1 [file vetsci-12-00960-s001.zip › vetsci-3703718-supplementary.pdf]

Analysis of antimicrobial residues and resistance profiles of *Escherichia coli* and *Enterococcus spp.* in lagoon water from California dairies

Siqi Wang [1], Sharif S Aly [1,2] Essam Abdelfattah [1,3*], Pius Ekong [2], David B Sheedy [2], Wagdy ElAshmawy [2], Betsy M Karle [4], Randi Black [5], Deniece R Williams [2], Pramod Pandey [1] and Emmanuel Okello [1,2,*]

[1] Department of Population Health and Reproduction, School of Veterinary Medicine, University of California, Davis, Davis, CA, United States.

[2] Veterinary Medicine Teaching and Research Center, School of Veterinary Medicine, University of California Davis, Tulare, CA 93274, USA

[3] Department of Animal Hygiene and Veterinary Management, Faculty of Veterinary Medicine, Benha University, Al Qalyubiyah, Egypt

[4] Cooperative Extension, Division of Agriculture and Natural Resources, University of California, Orland, CA, USA

[5] Cooperative Extension, Division of Agriculture and Natural Resources, University of California, Santa Rosa, CA, USA

* Correspondences: Essam Abdelfattah: eabdelfattah@ucdavis.edu; Emmanuel Okello: eokello@ucdavis.edu

Supplementary Tables

Table S1. Percent of antimicrobial resistance in *Escherichia coli* isolated from lagoon samples of 9 California dairies from 2018 to 2019.

***E. coli* (N=75)**				
**Antimicrobial agent**	**MIC Breakpoints(ug/mL)**	**% Resistant**	**SE**	**95%CI**
Ampicillin	≥16	18.67	6.73	7.63, 38.95
Ceftiofur	≥4	4.00	1.93	1.29,11.72
Danofloxacin	≥0.5	2.67	1.73	0.59, 11.28
Enrofloxacin	≥0.5	2.67	1.73	0.59, 11.28
Florfenicol	≥4	96.00	2.01	87.77, 98.77
Gamithromycin	≥8	92.00	1.87	86.47, 95.39
Gentamicin	≥8	1.33	1.32	0.13, 12.05
Neomycin	≥8	5.33	2.89	1.48, 17.44
Spectinomycin	≥64	1.33	1.32	0.13, 12.05
Sulphadimethoxine	>256	33.33	4.11	24.61, 43.37
Tetracycline	≥4	22.67	4.67	13.69, 35.14
Tiamulin	≥32	100.00	0.00	.
Tildipirosin	≥8	29.33	5.05	19.14, 42.13
Tilmicosin	≥16	100.00	0.00	.
Trimethoprim-sulfamethoxazole	> 2/38	1.33	1.32	0.13, 12.05
Tulathromycin	≥32	5.33	2.82	1.52, 16.95
Tylosin	≥16	100.00	0.00	.

Table S2. Percent of antimicrobial resistance in *Enterococcus spp. and Streptococcus spp.* isolated from lagoon samples of 9 California dairies from 2018 to 2019.

ES (N=82)				
**Antimicrobial agent**	**MIC Breakpoints(ug/mL)**	**% Resistant**	**SE**	**95%CI**
Ampicillin	≥16	4.88	1.91	1.95, 11.70
Florfenicol	≥4	95.12	1.95	88.08, 98.09
Gamithromycin	≥8	51.22	5.15	39.49, 62.82
Penicillin	≥8	8.54	2.38	4.42, 15.86
Tetracycine	≥4	50.00	6.34	35.79, 64.21
Tildipirosin	≥8	97.56	1.65	89.04, 99.50
Tilmicosin	≥16	98.78	1.24	88.33, 99.88
Tulathromycin	≥32	45.12	5.09	33.86, 56.91
Tylosin	≥16	30.49	6.90	17.15, 48.18

Table S3. Final logistic regression models for the association between drug residue and AMR in *Escherichia coli* isolated from lagoon samples of 9 California dairies from 2018 to 2019 (n= 75 samples).

**Outcome**[1]	**Predictor**[2]	**Coefficient**	**Robust SE**	**P Value**	**OR**	**95% CI**	
						**Lower**	**Upper**
Ampicillin[3]	Sulfamethoxazone	1.33	0.77	0.08	3.79	0.84	17.18
	Region (NCA)	0.49	0.62	0.43	1.63	0.49	5.48
Ceftiofur[4]	Tetracycline	-0.20	1.73	0.89	0.82	0.06	12.10
	Season (Spring)	0.41	1.60	0.80	1.50	0.07	34.25
	Region (NCA)	1.03	1.04	0.32	2.81	0.36	21.75
Danofloxacin[5]	Tilmicosin	-0.15	1.31	0.02	0.04	0.00	0.56
Enrofloxacin[6]	Tilmicosin	-0.15	1.31	0.02	0.04	0.00	0.56
Florfenicol[7]	Penicillin	-3.43	1.87	0.07	0.03	0.00	1.26
	Region (NCA)	-0.26	1.37	0.85	0.77	0.05	11.35
Gamithromycin[8]	Penicillin	-1.94	1.86	0.30	0.14	0.00	5.53
	Season (Spring)	0.93	0.95	0.33	2.53	0.39	16.32
	Region (NCA)	-1.15	0.64	0.07	0.32	0.09	1.11
	Region (NSJV)	-0.10	0.75	0.89	0.90	0.21	3.93
Neomycin[9]	Sulfamethoxazone	0.80	1.52	0.60	2.23	0.11	44.16
	Region (NCA)	1.55	0.89	0.08	4.70	0.82	26.93
Sulphadimethoxine[10]	Sulfamethoxazone	-0.12	0.68	0.86	0.89	0.23	3.38
	Region (NCA)	0.37	0.55	0.50	1.45	0.50	4.26
	Region (NSJV)	-0.22	0.31	0.48	0.80	0.43	1.48
	Sulfamethoxazone*Region (NCA)	1.32	1.04	0.24	3.44	0.45	26.48

	Sulfamethoxazone*Region (NSJV)	2.01	0.85	0.02	7.50	1.43	39.34
Tetracycline[11]	Sulfamethoxazone	0.03	0.65	0.96	1.03	0.29	3.67
	Season (Spring)	-0.61	0.54	0.26	0.54	0.19	1.57
	Region (NCA)	0.46	0.40	0.25	1.58	0.72	3.47
	Region (NSJV)	-1.50	0.85	0.08	0.22	0.04	1.19
Tildipirosin[12]	Tilmicosin	-2.69	1.12	0.02	0.07	0.01	0.61
	Region (NCA)	1.07	0.37	<0.01	2.90	1.41	5.98
	Region (NSJV)	-0.97	0.47	0.04	0.38	0.15	0.96
Tulathromycin[13]	Sulfamethoxazone	-0.43	0.91	0.64	0.65	0.11	3.86
	Season (Spring)	0.85	0.91	0.35	2.35	0.40	13.90
	Region (NCA)	-0.93	1.06	0.38	0.39	0.05	3.16

[1] Specific drug residues were perfectly correlated with susceptibility or resistance, such that presence of the residue resulted in all the isolates from these samples being either all susceptible or all resistant. Presence of penicillin residue, tetracycline residue, sulfamethoxazone residue, florfenicol residue or tilmicosin residue were all completely correlated with resistance or susceptibility of *E. coli* against gentamycin, spectinomycin or trimethoprim-sulfamethoxazole. The outcomes of AMR in *E. coli* were 100% resistant to tiamulin, tilmicosin or tylosin, so that these models could not be specified.

[2] Best fitting models, using the lowest Akaike Information Criterion estimate, were identified after offering each of the predictor variables for penicillin, tetracycline, sulfamethoxazole, florfenicol, or tilimicosin residues. Models were adjusted for confounders and effect-modification between residue resulting in the best fitting model by season and region. Season Fall and Region GSCA were used as references.

[3] Absence of florfenicol residue (n=1) was completely correlated with susceptibility of *E. coli* against ampicillin. Region NSJV (n=16) was completely correlated with susceptibility of *E. coli* against ampicillin.

[4] Presence of penicillin residue (n=3) was completely correlated with susceptibility of *E. coli* against ceftiofur. Absence of sulfamethoxazone (n=52), florfenicol (n=1) or tilmicosin residue(n=4) was completely correlated with susceptibility of *E. coli* against ceftiofur. Region NSJV (n=16) was completely correlated with susceptibility of *E. coli* against ceftiofur.

[5] Absence of penicillin residue (n=2) was completely correlated with resistance of *E. coli* against danofloxacin. Presence of sulfamethoxazone (n=2), florfenicol (n=2) or tilmicosin residue (n=2) was completely correlated with resistance of *E. coli* against danofloxacin. Season Spring (n=2) was completely correlated with susceptibility of *E. coli* against danofloxacin. When adjusted for Region, convergence of the model was not achieved.

[6] Absence of penicillin residue (n=2) was completely correlated with resistance of *E. coli* against enrofloxacin. Presence of sulfamethoxazone (n=2), florfenicol (n=2) or tilmicosin residue (n=2) was completely correlated with resistance of *E. coli* against enrofloxacin. Season Spring (n=2) was completely correlated with susceptibility of *E. coli* against enrofloxacin. When adjusted for Region, convergence of the model was not achieved.

[7] Absence of florfenicol (n=2) or tilmicosin residue (n=8) was completely correlated with resistance of *E. coli* against florfenicol. Season Spring (n=44) was completely correlated with resistance of *E. coli* against florfenicol. Region NSJV (n=16) was completely correlated with resistance of *E. coli* against florfenicol.

[8] Absence of florfenicol (n=2) or tilmicosin residue (n=8) was completely correlated with resistance of *E. coli* against gamithromycin.

[9] Presence of penicillin residue (n=3) was completely correlated with susceptibility of *E. coli* against neomycin. Absence of florfenicol residue (n=1) was completely correlated with susceptibility of *E. coli* against neomycin. Region NSJV (n=16) was completely correlated with susceptibility of *E. coli* against neomycin.

[10] Absence of florfenicol (n=1) was completely correlated with susceptibility of *E. coli* against sulphadimethoxine.

[11] Presence of penicillin residue (n=3) was completely correlated with susceptibility of *E. coli* against tetracycline. Absence of florfenicol residue (n=1) was completely correlated with susceptibility of *E. coli* against tetracycline.

[12] Presence of penicillin residue (n=3) was completely correlated with susceptibility of *E. coli* against tildipirosin. Absence of florfenicol residue (n=1) was completely correlated with resistance of *E. coli* against tildipirosin.

[13] Presence of penicillin residue (n=3) was completely correlated with susceptibility of *E. coli* against tulathromycin. Absence of florfenicol residue (n=1) or tilmicosin residue (n=4) was completely correlated with susceptibility of *E. coli* against tulathromycin. Region NSJV (n=16) was completely correlated with susceptibility of *E. coli* against tulathromycin.

Table S4. Final logistic regression models for the association between drug residue and AMR in *Enterococcus spp. / Streptococcus spp.* isolated from lagoon samples of 9 California dairies from 2018 to 2019 (n= 82 samples).

Outcome[1]	Predictor[2]	Coefficient	Robust SE	P Value	OR	95% CI	
						Lower	Upper
Ampicillin[3]	Sulfamethoxazone	0.84	1.05	0.43	2.31	0.29	18.41
	Region (NSJV)	-0.32	0.83	0.70	0.73	0.14	3.71
Florfenicol[4]	Tetracycline	0.03	1.13	0.98	1.04	0.11	9.43
Gamithromycin[5]	Tilmicosin	-1.40	1.03	0.17	0.25	0.03	1.86
	Season (Spring)	0.53	0.46	0.25	1.70	0.69	4.14
Penicillin[6]	Sulfamethoxazone	0.83	1.11	0.46	2.30	0.26	20.31
	Season (Spring)	1.90	1.10	0.09	6.69	0.77	58.35
Tetracycine[7]	Sulfamethoxazone	1.41	0.42	<0.01	4.11	1.79	9.44
	Season (Spring)	1.64	0.34	<0.01	5.17	2.67	10.00
	Sulfamethoxazone*Season (Spring)	-1.71	0.80	0.03	0.18	0.04	0.87
Tildipirosin[8]	Sulfamethoxazone	-0.73	1.65	0.66	0.48	0.02	12.31
Tulathromycin[9]	Tetracycline	0.48	0.58	0.41	1.62	0.52	5.05
	Season (Spring)	1.14	0.48	0.02	3.12	1.12	8.03
	Region (NCA)	-0.69	0.44	0.12	0.50	0.21	1.19
	Region (NSJV)	-1.28	0.34	<0.01	0.28	0.14	0.54
Tylosin[10]	Sulfamethoxazone	0.84	0.62	0.17	2.32	0.69	7.81
	Season (Spring)	1.54	0.41	<0.01	4.67	2.08	10.47

[1] Specific drug residues were perfectly correlated with susceptibility or resistance, such that presence of the residue resulted in all the isolates from these samples being either all susceptible or all resistant. Presence of penicillin residue, tetracycline residue, sulfamethoxazone residue, florfenicol residue or tilmicosin residue were all completely correlated with resistance or susceptibility of ES against tilmicosin.

[2] Best fitting models, using the lowest Akaike Information Criterion estimate, were identified after offering each of the predictor variables for penicillin, tetracycline, sulfamethoxazole, florfenicol, or tilimicosin residues. Models were adjusted for confounders and effect-modification between residue resulting in the best fitting model by season and region. Season Fall and Region GSCA were used as references.

[3] Presence of penicillin residue (n=2) was completely correlated with susceptibility of ES against ampicillin. Absence of tetracycline residue (n=20), florfenicol residue (n=1) or tilmicosin residue (n=4) was completely correlated with susceptibility of ES against ampicillin. Region NCA (n=28) was completely correlated with susceptibility of ES against ampicillin. Season Fall (n=37) was completely correlated with susceptibility of ES against ampicillin.

[4] Presence of penicillin residue (n=2) was completely correlated with resistance of ES against florfenicol. Absence of florfenicol residue (n=1) or tilmicosin residue (n=4) was completely correlated with resistance of ES against florfenicol.

[5] Presence of penicillin residue (n=2) was completely correlated with susceptibility of ES against gamithromycin. Absence of florfenicol residue (n=1) was completely correlated with resistance of ES against gamithromycin.

[6] Presence of penicillin residue (n=2) was completely correlated with susceptibility of ES against penicillin. Absence of tetracycline residue (n=20), florfenicol residue (n=1) or tilmicosin residue (n=4) was completely correlated with susceptibility of ES against penicillin.

[7] Presence of florfenicol residue (n=1) was completely correlated with resistance of ES against tetracycline.

[8] Presence of penicillin residue (n=2) was completely correlated with susceptibility of ES against tildipirosin. Absence of tetracycline residue (n=20), florfenicol residue (n=1) or tilmicosin residue (n=4) was completely correlated with resistance of ES against tildipirosin.

[9] Presence of penicillin residue (n=2) was completely correlated with susceptibility of ES against tulathromycin. Absence of florfenicol residue (n=1) was completely correlated with susceptibility of ES against tulathromycin.

[10] Presence of penicillin residue (n=2) was completely correlated with susceptibility of ES against tylosin. Absence of florfenicol residue (n=1) was completely correlated with susceptibility of ES against tylosin.
